# Quantification of bowel ischaemia using real-time multispectral Single Snapshot Imaging of Optical Properties (SSOP)

**DOI:** 10.1007/s00464-022-09764-z

**Published:** 2022-11-28

**Authors:** María Rita Rodríguez-Luna, Nariaki Okamoto, Lorenzo Cinelli, Luca Baratelli, Silvère Ségaud, Adriana Rodríguez-Gómez, Deborah S. Keller, Elham Zonoobi, Elisa Bannone, Jacques Marescaux, Michele Diana, Sylvain Gioux

**Affiliations:** 1 Research Institute Against Digestive Cancer (IRCAD), 1, place de l’Hôpital, 67000 Strasbourg Cedex, France; 2 University of Strasbourg, ICube Laboratory, Strasbourg, France; 3 Department of Gastrointestinal Surgery, San Raffaele Hospital IRCCS, Milan, Italy; 4 Department of Pathology, San Angel Inn Sur Hospital, Mexico City, Mexico; 5 Marks Colorectal Surgical Associates, Lankenau Medical Center, Main Line Health, Wynnewood, PA USA; 6 Edinburgh Molecular Imaging Ltd. (EMI), Edinburgh, EH16 4UX UK; 7 Department of Surgery, Leiden University Medical Center, 2300 RC Leiden, The Netherlands; 8 Department of General and Pancreatic surgery – The Pancreas Institute, University of Verona, Verona, Italy; 9 Intuitive Surgical Sàrl, Aubonne, Switzerland

**Keywords:** Single snapshot imaging of optical properties, Image-guided surgery, Diffuse optical imaging, Tissue perfusion, Anastomotic leak

## Abstract

**Background:**

Single snapshot imaging of optical properties (SSOP) is a relatively new non-invasive, real-time, contrast-free optical imaging technology, which allows for the real-time quantitative assessment of physiological properties, including tissue oxygenation (StO2). This study evaluates the accuracy of multispectral SSOP in quantifying bowel ischaemia in a preclinical experimental model.

**Methods:**

In six pigs, an ischaemic bowel segment was created by dividing the arcade branches. Five regions of interest (ROIs) were identified on the bowel loop, as follows: ROI 1: central ischaemic; ROI 2: left marginal; ROI 3: left vascularised; ROI 4: right marginal; and ROI 5: right vascularised. The Trident imaging system, specifically developed for real-time tissue oxygenation imaging using SSOP, was used to image before (T0) and after ischaemia induction. Capillary and systemic lactates were measured at each time point (T0, T15, T30, T45, T60), as well as StO2 values acquired by means of SSOP (SSOP-StO2).

**Results:**

The mean value of SSOP-StO2 in ROI 1 was 30.08 ± 6.963 and was significantly lower when compared to marginal ROIs (ROI 2 + ROI 4: 45.67 ± 10.02 *p =  <* 0.0001), and to vascularised ROIs (ROI 3 + ROI 5: 48.08 ± 7.083 *p =  <* 0.0001).

SSOP-StO2 was significantly correlated with normalised lactates *r =* − 0.5892 *p <* 0.0001 and with histology *r =*− 0.6251 *p = *0.0002.

**Conclusion:**

Multispectral SSOP allows for a contrast-free accurate assessment of small bowel perfusion identifying physiological tissue oxygenation as confirmed with perfusion biomarkers.

**Supplementary Information:**

The online version contains supplementary material available at 10.1007/s00464-022-09764-z.

Anastomotic leak (AL) is a major complication in digestive surgery. It is accountable for considerable morbidity and mortality [1, 2]. The AL incidence is particularly high following oesophageal (20–35%) and colorectal resections (4–19%) [3], and in cases which require extraperitoneal anastomosis [4]. The pathophysiology of AL is multifactorial and includes patient non-modifiable factors such as associated medical conditions, nutritional score, and oncological status including neoadjuvant treatment. On the other hand, there are surgical modifiable factors, which influence the incidence of AL such as the transection level ensuring optimal perfusion.

Adequate perfusion is a key component of the anastomotic healing process. However, an intraoperative evaluation of perfusion is mostly based on often unreliable clinical criteria, namely serosal discolouration, blood flow from the marginal artery, and pulsatile bleeding at the cut edge of the bowel [5]. As a result, there is a compelling clinical need to develop intraoperative technologies which enhance the surgeon’s ability to quantify and identify transection and anastomotic sites.

Over recent years, near-infrared (NIR) fluorescence imaging (FI) based on indocyanine green (ICG) has been extensively studied as a tool to provide an enhanced evaluation of bowel perfusion in real time [6]. Promising results have been demonstrated in several prospective clinical trials in which ICG-FI improves surgical outcomes by decreasing the incidence of AL [7, 8].

However, the slow, yet consistent diffusion of the fluorophore from perfused to non-perfused areas, as well as the lack of quantification of the fluorescence signal may lead to errors in the identification of marginal zones. Additionally, the estimation of perfusion using fluorescence angiography (FA) is limited to perfusion-only metrics (as opposed to the functional tissue status) and necessitates the injection of an exogenous agent [5].

In parallel to exogenous fluorescence, non-invasive contrast-free (i.e. endogenous) real-time optical imaging technologies have been developed. They are promising tools since they allow for an adequate surgical workflow without the constraints of administering external chemical compounds to the human body. Consequently, the clinical translation of endogenous optical imaging devices is strongly accelerating and being disseminated.

In order to obtain physiological local information, most devices used are based on NIR spectroscopy, such as hyperspectral (HSI) and multispectral (MSI) imaging technologies [9–11]. Spectroscopic imaging allows for the non-contact reading of StO2 with high spatial resolution and with a large field of view (> 15 by 15 cm^2^), thereby improving intraoperative decision-making by adding quantitative information.

Single snapshot of imaging optical properties (SSOP) has recently been developed [12]. It is a contrast-free, real-time, non-invasive optical imaging technique, which allows to evaluate physiological tissue properties. SSOP is based on the well-known diffuse optimal imaging method named spatial frequency domain imaging (SFDI), which uses light propagation models to quantify optical properties discriminating physiological contrast from tissue constituents [13]. As a result, multispectral SSOP can determine tissue oxygenation (StO2) by computing the fraction of oxygenated over total haemoglobin in real time. It has proven to be an efficient method since it allows to reduce the number of image acquisitions to a single frame, which is very convenient in the surgical setting, hence allowing for a real-time video-rate imaging of StO2 and preventing from workflow disruption [14]. The performances of multispectral SFDI and SSOP have previously been evaluated both ex vivo and in vivo and they have proven to achieve a high accuracy (i.e. less than a 10% error) [15–17].

The aim of this experimental study was to preclinically evaluate the accuracy of multispectral SSOP in quantifying bowel perfusion using normalised capillary lactate as a validated perfusion biomarker.

## Methods

### Animal models

A total of 6 adult swine (*Sus scrofa domesticus*, ssp. Large white, mean weight: 40 kg) were involved in this non-survival study. The present experimental study is part of the QuantSURG (Quantitative Surgical Guidance for Colorectal Surgery) project, which received full approval from the local Ethical Committee on Animal Experimentation (ICOMETH No. 038.2019.01.121) and from the French Ministry of Superior Education and Research (MESR) under the following reference: APAFIS #20,819–2,019,052,411,591,088 v3. All animals used in the experimental laboratory were managed according to French laws for animal use and care and according to the directives of the European Community Council (2010/63/EU) and ARRIVE guidelines [18]. The animals were fasted for 24 h with free access to water before surgery. Animals were premedicated, 10 min before surgery with an intramuscular injection of ketamine (20 mg/kg) and azaperone (2 mg/kg) (Stresnil; Janssen-Cilag, Belgium). Intravenous propofol (3 mg/kg) combined with rocuronium (0.8 mg/kg) were used for induction. Anaesthesia was maintained with 2% isoflurane. At the end of the procedures, pigs were sacrificed with an intravenous injection of a lethal dose of potassium chloride.

### SSOP imaging

Single snapshot imaging of optical properties (SSOP) is based on the projection of spatially modulated patterns of light on the sample and on the acquisition with a camera of the diffused back reflected light. SSOP was developed as a real-time implementation of more time-consuming spatial frequency domain imaging (SFDI) methods, which require several frames for the extraction of the optical properties of tissues. Indeed, in a standard SFDI workflow, two different spatial frequency (f_x_) profiles (e.g. f_x_ = 0 mm^−1^, f_x_ = 0.2 mm^−1^) with three-phase shifts for each of them are projected on the sample surface, allowing for the demodulation of the signal into its DC and AC components [19]. Differently, SSOP requires only a single high-frequency pattern to be projected on the tissue, thanks to a Fourier’s domain filtering approach for the demodulation step [12, 15, 17], thereby reducing from 6 to 1 the number of frames required for the acquisition and allowing for a real-time capability of the imaging system. Further to the demodulation, both SFDI and SSOP share the same workflow starting from a calibration step involving the measurement of a calibration phantom with known optical properties. The measurements of this tissue-mimicking phantom allow to compute the diffuse reflectance maps for the sample, i.e. R_DC_ (f_x_ = 0 mm^−1^) and R_AC_ (f_x_ = 0.2 mm^−1^). Finally, a Monte Carlo-based lookup table (MCLUT) algorithm allows to retrieve the optical properties of the sample (i.e. absorption and reduced scattering coefficients) for each pixel of the image [20]. The natural consequences of the single frame approach are a degradation in image quality and the presence of edge artefacts. Nevertheless, significant improvements have been achieved in recent years by first optimising the filtering technique [21] and by adopting deep neural network approaches for the demodulation [15, 17]. Additionally, the latest developments on SSOP also account for a tridimensional profile correction of the sample in order to reduce the quantification error of SSOP associated with a variation of light intensity across non-flat sample surfaces [15]. Further information regarding SFDI is available as follows: http://opensfdi.org.

### Deep learning method for SSOP

For this study, the latest SSOP deep learning-based approach has replaced the standard Fourier’s domain filtering technique to improve the overall image quality, as described by Aguénounon et al. [15]. In brief, two dedicated convolutional neural networks (CNNs) based on a U-Net architecture were used for the extraction of the modulation amplitude of the signal for each spatial frequency, and for the profilometry analysis of the surface profile of the sample. Both networks were trained using high-quality images obtained with SFDI acquisition sequences (with 7-phase shifts instead of 3 in order to enhance quantification accuracy and image quality) and optimised for efficient and low-cost computation performances, allowing for a real-time capability and achieving high visual quality optical property quantification for up to 1-megapixel images. The training dataset consisted of a total of 200 high-quality images divided into *n = *40 images of tissue-mimicking silicone phantoms with different optical properties ranging from μ_a_ = 0.005 to 0.05 mm^− 1^ for absorption and from μ_s_’ = 0.5 to 3 mm^−1^ for reduced scattering; *n = *52 images of hands from different Caucasian men and women in various configurations; *n = *108 images from ex vivo and in vivo swine organs in several orientations (stomach, small bowel, colon, kidney, pancreas, liver, and spleen). Additional information regarding SSOP and its deep learning implementation (code and sample data) are available as follows: https://healthphotonics.org/ressources/sfdi-resources/.

### Trident imaging system

The Trident imaging system is based on a digital micromirror device (DMD, Vialux GmbH, Chemnitz, Sachsen, Germany) to project structured light in the form of sinusoidal patterns at a 45 ± 5 cm working distance. The projection system is fibre-coupled to a dedicated, custom-made class 3R high-power laser source composed of laser diodes (LDX Optronics, Maryville, TN, United States), which is used to project patterns at 2 wavelengths (i.e. 665 nm and 860 nm) for oxygen saturation multispectral measurements [22]. A white high-power LED lamp (FTIII24017-12, Fiberoptics Technology Inc., Pomfret Center, CT, United States) is also used to illuminate the surgical field to provide anatomical visualisation specifically filtered to prevent impeding oxygen saturation measurements. The imaging head is built with three CMOS cameras (JAI GO-5000 M-USB, JAI Ltd., Kanagawa, Japan, PCO.edge 4.2, Excelitas PCO GmbH, Kelheim, Germany, JAI GO-5000C-USB, JAI Ltd., Kanagawa, Japan) sharing the same field of view of 15 by 15 cm^2^ for the collection of the NIR1 (665 nm), NIR2 (860 nm), and the RGB channels with a resolution of 1024 by 1280 pixels. In addition, low-pass and high-pass filters are used in the optical path to isolate the different wavelengths (Chroma Technology Corp., Bellows Falls, VT, United States). A pair of linear polarisers (PPL05C, Moxtek, Orem, UT, United States) in a crossed configuration are also used at the projection and imaging sides to reject the contribution from specular reflections at the surface of the sample. A silicone-based optical phantom (21 by 21 by 2 cm) with known optical properties (${\mu }_{a}=0.01 m{m}^{-1}$ and ${\mu }_{s}^{^{\prime}}=1.1 m{m}^{-1}$ at 665 nm, and ${\mu }_{a}=0.02 m{m}^{-1}$ and ${\mu }_{s}^{^{\prime}}=0.8 m{m}^{-1}$ at 860 nm) is used for the calibration of the Trident imaging system. The imaging workflow consists of the simultaneous projection of a high-frequency sinusoidal pattern (here ${f}_{x}=0.3 m{m}^{-1}$) at the two wavelengths (665 nm and 860 nm) followed by the three-channel acquisition of the images for the extraction of the oxygen saturation level of the tissue.

### Surgical set-up of pure ischaemia model

A central venous line was placed by means of an internal jugular vein dissection. A midline laparotomy was performed using electrocautery. A self-retaining retractor was then placed and a 10 cm small bowel loop was exposed in order to create bowel ischaemia by dividing arcade branches. Five regions of interest (ROIs) were marked with an interrupted suture in the antimesenteric border (ROI 1: central ischaemic; ROI 2: left marginal; ROI 3: left vascularised (2.5 cm from ROI 2); ROI 4: right marginal; ROI 5: right vascularised (2.5 cm from ROI 4)). The small bowel was imaged with the multispectral camera through SSOP before and after ischaemia at different time points (T0, T15, T30, T45, T60 min). The SSOP image acquisitions were performed in the dark (i.e. with the light turned off in the operating room), in order to reduce the risk of bias. Nevertheless, the system can also be operated in standard ambient light conditions via an adapted calibration routine.

### Surgical set-up of ischaemia/reperfusion model

To assess the accuracy of the Trident system in detecting oxygenation changes, we created a short ischaemia/reperfusion model. Same surgical principles were respected for laparotomy. Six 10 cm small bowel loops were exposed in order to create short bowel ischaemia by dividing the peritoneal layer of the mesentery with sufficient length to apply a bulldog clamp. Similarly, to the pure ischaemic model, five regions of interest (ROIs) were marked with an interrupted suture in the antimesenteric border (ROI 1: central ischaemic; ROI 2: left marginal; ROI 3: left vascularised (2.5 cm from ROI 2); ROI 4: right marginal; ROI 5: right vascularised (2.5 cm from ROI 4) in each loop. Small bowel loops were imaged with the Trident system through SSOP before, during clamping, and after declamping at the reperfusion phase (T0, and T10, and T15 min, respectively).

### Analysis of capillary and systemic lactates

At each time point, capillary lactates (mmol/L) were measured by means of a portable analyser (EDGE ®lactate analyser, ApexBio, Taipei, Taiwan, People’s Republic of China) by puncturing the bowel’s serosal side at each ROI. Lactate is the product of glycolysis and its accumulation reflects a lowered mitochondrial activity in the presence of reduced O2 concentration. This method has been described earlier in studies on the metabolic effect of bowel resection [23, 24]. Systemic lactates were also measured in venous blood retrieved from the central line and using the EPOC® Blood Analysis System (Siemens Healthineers, Erlangen, Germany), a portable blood analyser.

### Pathological examination

For the pure ischaemic model, full-thickness biopsies were retrieved at the end of the procedure, and specimens were fixed in a 4% formalin solution for at least 24 h. Four-μm-thick sections were cut from paraffin-embedded tissues and stained with haematoxylin and eosin. Biopsies were taken from each ROI after 60 min of ischaemia and one from a distal part before the creation of the ischaemic loop, which served as a control. A microscopic assessment was made (Leica 2000 LED, Leica Biosystems GmbH, Wetzlar, Germany) using Park/Chiu’s scoring system of intestinal ischaemic damage [25, 26].

### Statistical analysis

All statistical analyses were performed using the GraphPad Prism software for macOS (GraphPad Software, Inc., San Diego, CA, United States), version 9.1.1. Data are shown as mean and standard deviations (SD) unless otherwise indicated. Capillary lactates were normalised according to systemic lactates to reduce variability. In order to assess a possible correlation between normalised lactates and SSOP-StO2, a Pearson’s rank correlation coefficient was calculated. A Student’s t test was used to compare continuous variables after confirmation of a parametric distribution using the Kolmogorov-Smirnov normality test. A *p* value < 0.05 was considered statistically significant.

## Results

**Fig. 1:**
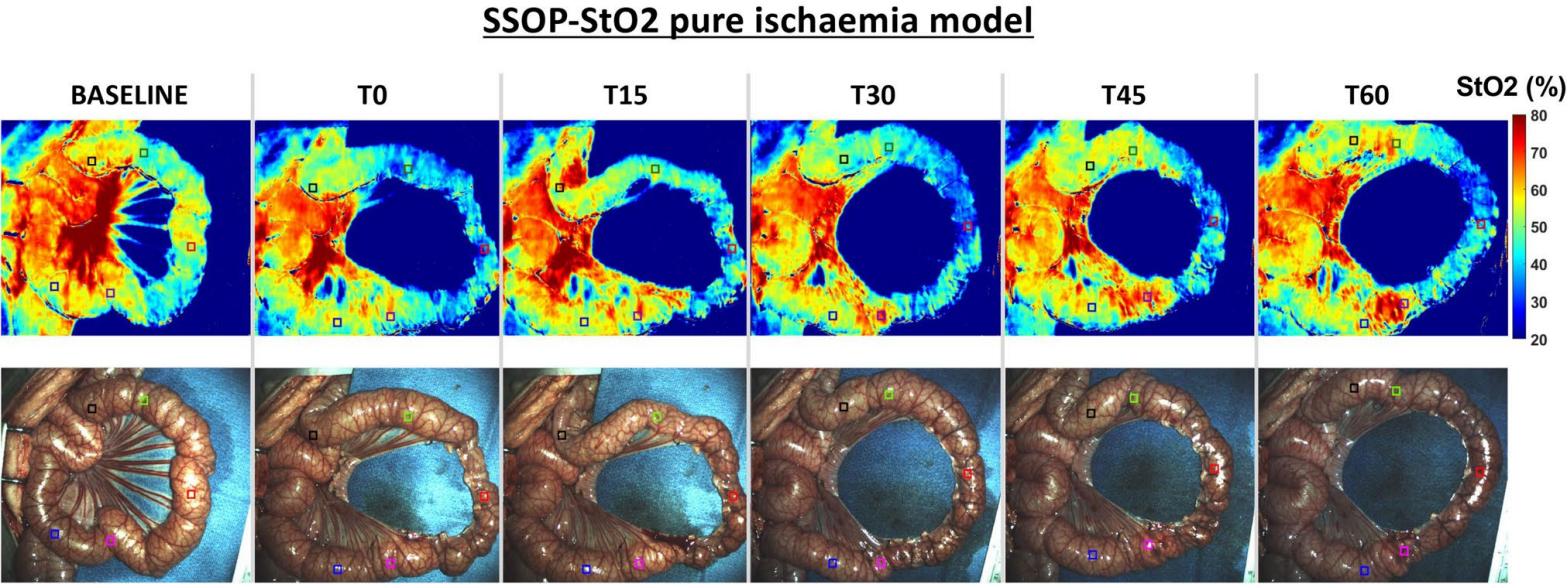
SSOP images showing StO2% during the full ischaemic period in the small bowel loop from baseline to T60, and corresponding true colour (RGB) images

**Fig. 2:**
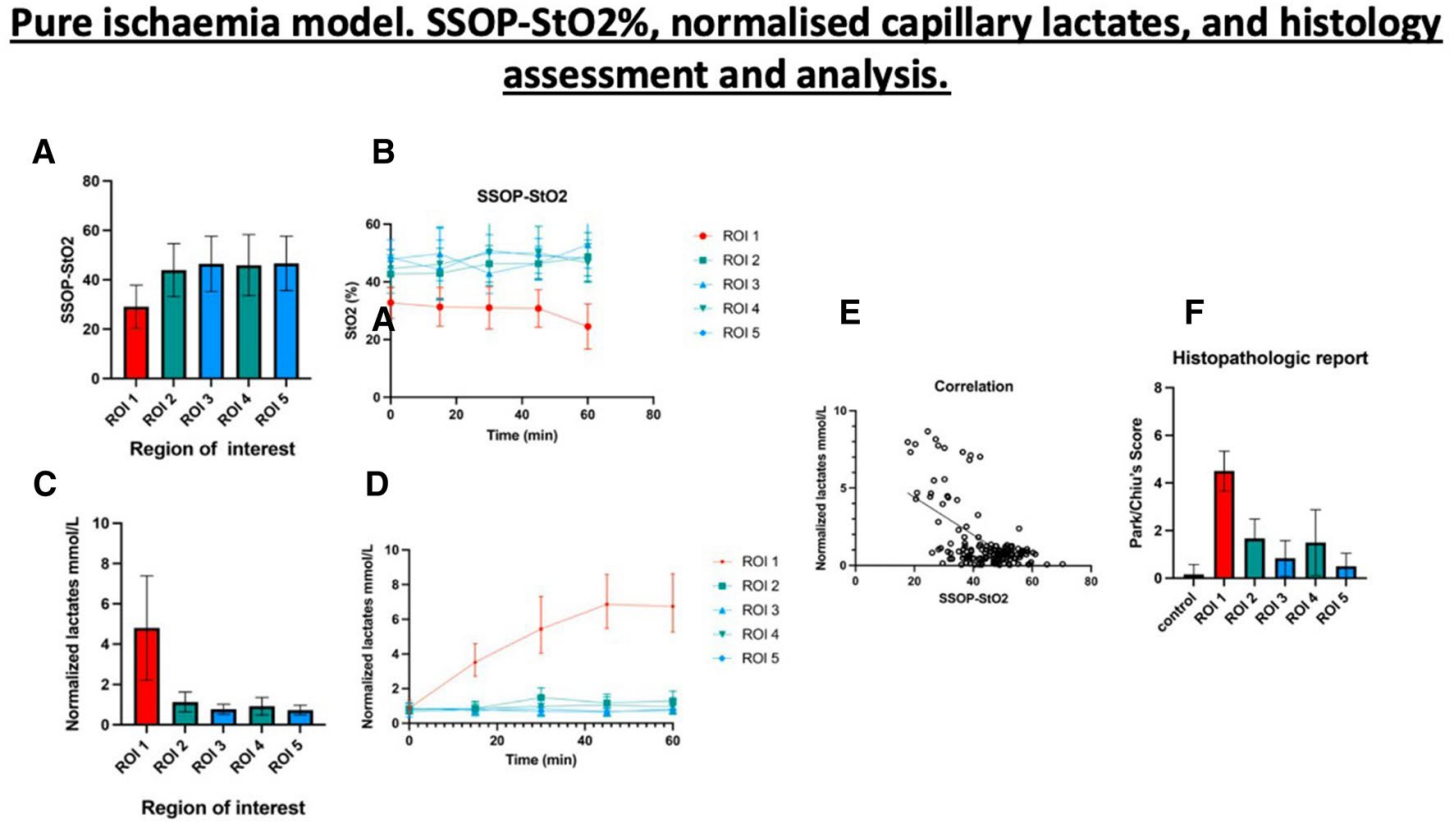
**A.** SSOP-StO2 values during 1 h of ischaemia: the mean value of SSOP-StO2 in ROI 1 was 30.08 ± 6.963 and significantly lower when compared to marginal ROIs (ROI 2 + ROI 4: 45.67 ± 10.02 *p =  <* 0.0001), and to vascularised ROIs (ROI 3 + ROI 5: 48.08 ± 7.083, *p =  <* 0.0001). Although SSOP-StO2 was higher in vascularised ROIs, it did not show any statistically significant difference (*p = *0.1298). **B**. Kinetics of StO2 cartography in each ROI. **C.** Normalised lactates (mmol/L) during 1 h of ischaemia: the mean value of normalised lactates in ROI 1 was 4.804  ± 2.591 and significantly higher when compared to marginal ROIs (ROI 2 + ROI 4: 1.026 ± 0.472, *p <* 0,0001), and to vascularised ROIs (ROI 3 + ROI 5: 0.749 ± 0.246, *p <* 0.0001). The difference between marginal and vascularised ROIs also showed a statistically significant difference *p <* 0.0001. **D.** Kinetics of normalised lactates (mmol/L). **E. **Pearson’s correlation analysis between normalised lactates and SSOP-StO2 in correspondence to all ROIs. **F**. Histopathological report: the mean Park/Chiu’s score at ROI 1 was 4.500 ± 0.8367 and significantly higher than marginal zones (ROI 2 and ROI 4: 1.583 ± 1.097, *p <* 0,0001) and vascularised (ROI 3 and ROI 5: 0.667 ± 0.650, *p <* 0.0001)

In the pure ischaemia model, the oxygenation parameter for each ROI was assessed using SSOP-StO2 (Fig. 1). The mean value of SSOP-StO2 in ROI 1 was 30.08% ± 6.963 and significantly lower when compared to marginal ROIs (ROI 2 + ROI 4: 45.67% ± 10.02, *p =  <* 0.0001), and to vascularised ROIs (ROI 3 + ROI 5: 48.08% ± 7.083, *p =  <* 0.0001). Although the SSOP-StO2 was higher in vascularised ROIs, this difference was not statistically significant (*p = *0.1298) (Fig. 2A and B).

The mean value of normalised lactates in ROI 1 was 4.804 mmol/L ± 2.591 and significantly higher when compared to marginal ROIs (ROI 2 + ROI 4: 1.026 mmol/L ± 0.472 *p <* 0.0001), and to vascularised ROIs (ROI 3 + ROI 5: 0.749 ± 0.246 mmol/L, *p <* 0.0001). The difference between marginal and vascularised ROIs also showed a statistically significant difference (*p <* 0.0001) (Fig. 2C and D).

The cumulative Pearson’s correlation analysis between normalised lactates and SSOP-StO2 was -0.5892, *p <* 0.0001 (Fig. 2E).

As for the histopathological confirmation, the mean Park/Chiu’s score at ROI 1 was 4.500 ± 0.8367 and was significantly higher than marginal zones (ROI 2 + ROI 4: 1.583 ± 1.097) *p <* 0.0001 and vascularised ROIs (ROI 3 + ROI 5: 0.667 ± 0.650), *p <* 0.0001 (Fig. 2F).

At T65, the Park/Chiu’s score correlation between SSOP-StO2 was *r =*− 0.6251, *p = *0.0002 and between normalised lactates was *r =* 0.7102, *p <* 0.0001.

**Fig. 3:**
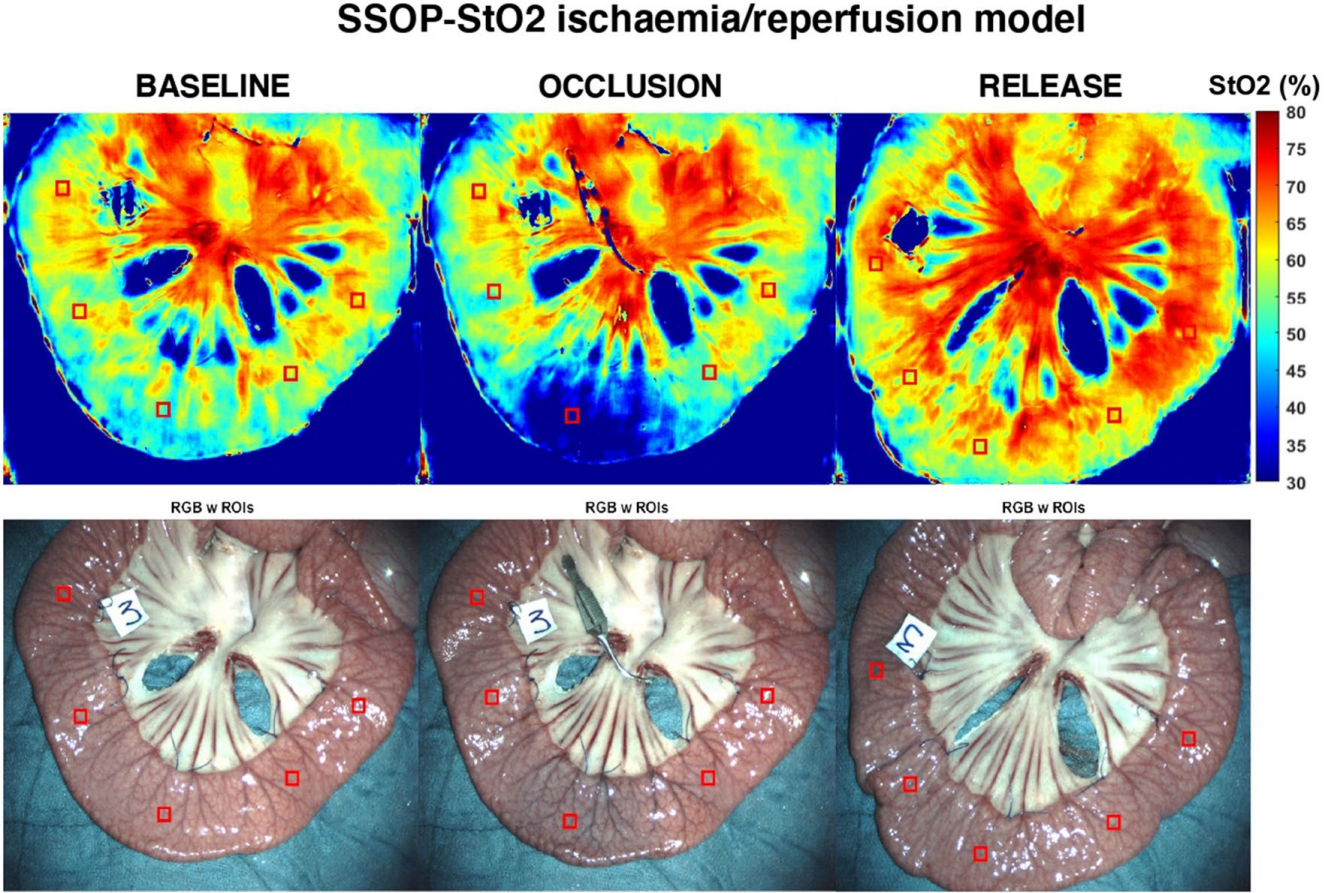
SSOP images showing SSOP-StO2% in the ischaemia/reperfusion small bowel model at baseline, occlusion, and release with the corresponding true colour (RGB) images (Color figure online)

**Fig. 4:**
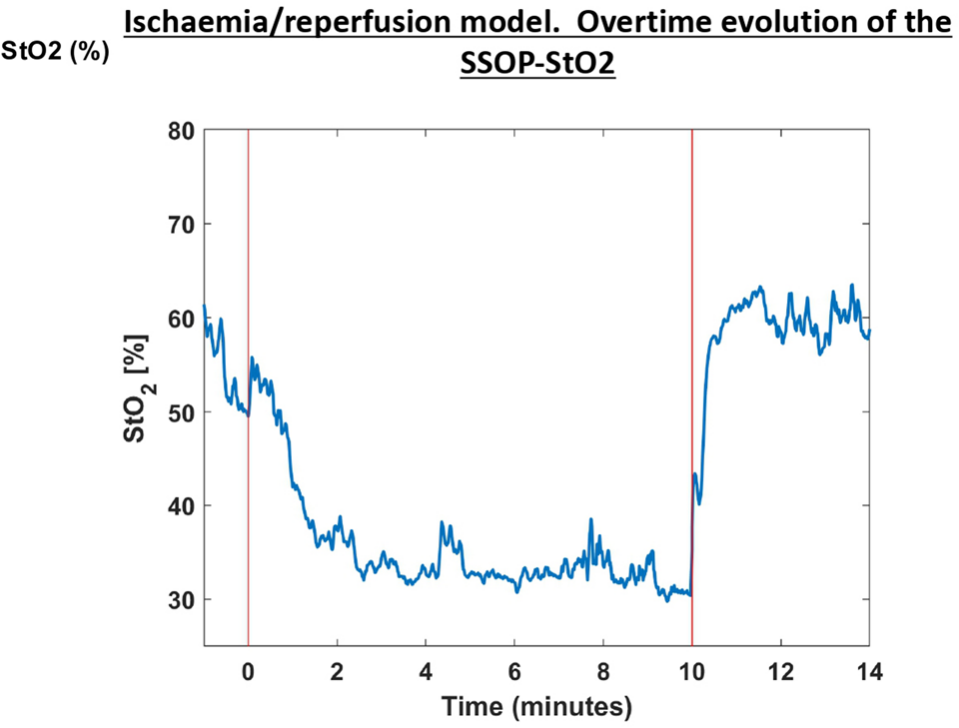
The graph shows the overtime evolution of the SSOP-StO2 parameter at baseline, occlusion, and reperfusion phases on ROI 1. Red bars represent clamping (T0) and declampling (T10), respectively. The SSOP-StO2 could precisely discriminate when the ischaemia started with a more evident decrease in 50% appreciated after 2 min of occlusion. Once the surgical clamp was released (second red bar), an improvement in saturation occurred

In the short ischaemia/reperfusion model, we found that ROI 1 has a mean 57.54 ± 5.35, 37.6 ± 4.12, and 62.40 ± 7.28 SSOP-StO2 at T0, T10, and T15, respectively. The difference in terms of SSOP-StO2 value from occlusion to reperfusion was statistically significant (*p = *0.00017) (Figs. 3 and 4) (Video 1). Marginal ROIs (ROI 2 + ROI 4) had a mean of 57.22 ± 5.61, 56.89 ± 5.49, and 60.93 ± 6.91 SSOP-StO2 at T0, T10, and T15, respectively, while vascularised ROIs (ROI 3 + ROI 5) had a mean of 62.53 ± 5.43, 59.71 ± 6.37, and 64.40 ± 6.80 SSOP-StO2 at T0, T10, and T15, respectively. In marginal ROIs (ROI 2 + ROI 4), the differences in terms of SSOP-StO2 from occlusion to reperfusion were not statistically significantly different (*p = *0.1511), similarly to vascularised ones (*p = *0.1167).

## Discussion

In the present experimental pure bowel ischaemia model, the Trident imaging system could provide accurate quantification of tissue StO2, which is a useful biomarker of tissue viability and is also correlated with a validated marker of tissue perfusion [24]. Capillary lactates have been substantially studied by our research group as a ground truth assessing NIR optical imaging technologies [9, 27].

Local bowel capillary lactates were normalised via systemic lactates to reduce the variability between animals. The high number of paired normalised capillary lactates and StO2 datasets (175) at different time points allowed to compute a negative correlation between those two parameters. Previously, our group reported a correlation between StO2 and lactates using HSI within similar ranges (*r =* − 0.7) [28].

Additionally, StO2 images from the Trident imaging system were also correlated with the Park/Chiu’s score which is based on histopathological assessment of all intestinal layers. Although it is well-known that the intestinal mucosa and submucosa are the first ones affected by impaired perfusion since they receive most of the mesenteric blood flow (70%), the muscularis and serosal layers can also be damaged during ischaemic periods [27]. Since the evaluation of the StO2 images was performed on the serosal side, histopathological repercussions in external layers were elements of interest. Nevertheless, this was a short-term ischaemia model (1 h), and transmural necrosis is mostly developed after longer ischaemic periods (6 h) [27]. In fact, the higher Park/Chiu’s score of 6 was found in one case only.

To verify the accuracy of the Trident system, we created the short ischaemia/reperfusion model in which the Trident system could detect the variation of StO2 as soon as the vascular clamp was applied and released.

Intraoperative perfusion assessment constitutes a critical issue during digestive surgery. In NIR-FA, ICG has so far been the most commonly used fluorophore to evaluate local microperfusion [29]. ICG-FA has been rapidly expanded by the increasing availability of optical imaging systems [30–32]. However, most ICG-FA optical systems are based on relative fluorescence intensity (FI) without considering diffusion, which can potentially lead to overestimation on non-perfused areas. Secondly, FI is inversely correlated to the source-to-target distance. This fact makes those structures closer to the source brighter than areas far away. To prevent any distance bias, distance standardisation between the NIR endoscope and the surgical field and/or the use of a reference calibration tool must be performed during image acquisition [33].

In order to overcome ICG-FA drawbacks, quantification methods which are irrespective of distance have been developed to obtain a perfusion cartogram based on the dynamic uptake of the fluorophore over time such as FLER [29] or Q-ICG [34, 35]. Although the use of quantitative methods offers a reproducible and robust solution, they have not been extensively adopted at present and there is still no standardised approach regarding its use as reported in the IHU‐IRCAD‐EAES EURO‐FIGS registry [36].

As compared to FA, endogenous imaging methods including SSOP can provide a greater amount of quantitatively significant data by characterising several tissue components including StO2, which reflects intracellular metabolic changes induced by ischaemia. HSI is an eminent well-known example of endogenous imaging. Its accuracy in assessing perfusion during oesophagectomy has been studied in 22 patients by discriminating between gastric conduits with and without laparoscopic ischaemic preconditioning (StO2 66 *vs.* 78%, *p = *0.03) [37]. In colorectal surgery, HSI has been compared between to FA, showing comparable results in detecting the optimal demarcation line [38].

Although HSI is an efficient technology, it does not allow for a video-rate acquisition and the large datasets require postprocessing algorithms to discriminate features according to spectral curves. To overcome this limitation, our group created the concept of HYPerspectral Enhanced Reality (HYPER) which had promising results in the preclinical setting [9]. However, HYPER requires image superimposition using augmented reality onto real-time videos to achieve real-time intraoperative guidance, making its use currently limited [3].

At present, SSOP is a more adapted surgical navigation system method since it allows for a video rate (> 25 frames per second), and a wide-field (> 100 cm^2^), non-invasive, quantitative multispectral characterisation of metabolic properties through a single frame acquisition [19, 39, 40].

A strong point is that SSOP is based on SFDI that in one of the first in-human pilot trials has proven to be accurate in assessing the oxygenation of microsurgical deep inferior epigastric perforator flaps during reconstructive breast surgery [41]. However, standard SFDI is time-consuming since it requires the acquisition of several images (in this case, 6 images), being inconvenient for the surgical workflow.

To address such constraints, SSOP was developed as an enhanced fast optical intraoperative navigation tool. However, with the conventional process using image reduction, SSOP initially suffered from degraded visual quality as compared to the 7-phase SFDI. Artificial intelligence has been used to overcome such issues. A Graphics Processing Units (GPU)-accelerated deep learning (DL) as a processing technology has been previously described. SSOP GPU-accelerated DL evaluation of in vivo human hands and ex vivo animal organs showed superiority in terms of image resolution and degradation as compared to a classical SSOP-filtering approach. The DL processing methodology has shown errors as low as 7.5 ± 2.7% [15].

In this study, the Trident imaging system uses SSOP GPU-accelerated DL using CCNs and allows for a non-contrast physiological real-time multispectral assessment in an in vivo one-hour ischaemic model (Fig. 1). Additionally, the use of artificial intelligence helped to maintain high image quality comparable to the 7-phase SFDI. The promising results presented in this article of SSOP-StO2 can be potentially expanded into other fields for surgical guidance.

This study relies on a robust methodology. The limited number of animals represents a constraint since 6 pigs were only studied, in which SSOP-StO2 and capillary lactates standard deviations between marginal and vascularised ROIs were high. Although SSOP-StO2 was high in vascularised compared to marginal ROIs, it was not statistically significant. Less variability could be encountered if sample size is increased during future experiments. Another downside of the present study lies in the postprocessing time GPU-accelerated DL and in its non-survival design, which does not make it possible to evaluate the impact of multispectral SSOP on anastomotic healing. As a result, the next sensible step will be to evaluate multispectral SSOP performance in a survival anastomotic model.

Since the current SSOP-based optical imaging system is designed for open settings, a potential improvement making way for clinical translation relies on hardware refinement. Presently, our group is strongly considering this parameter to enhance such an optical imaging technology.

In conclusion, based on standardised metabolic biomarkers, SSOP-StO2 allows for a contrast-free assessment of small bowel perfusion, identifying physiological tissue properties.

## Supplementary Information

Below is the link to the electronic supplementary material.

Supplementary file1 (MP4 132491 kb)

## Data Availability

The data that support the findings of this study are available from the corresponding author upon reasonable request.
